# Correspondence

**Published:** 1869-10

**Authors:** 


### Correspondence.

*To the Editor of the Buffalo Medical and Surgical Journal:*

DEAR SIR,—I, yesterday, received the September number (1869) of your Journal, postmarked Buffalo, N. Y. To whom I am indebted for this act of courtesy, I know not; but, as a *private* letter of mine to Professor JAMES P. WHITE is introduced on page 66 by that gentleman, and a criticism follows it by yourself, I infer that my thanks are either due to him or to you.

May I enquire what was the object in publishing that letter? Was it truly with the "*belief that every important advance in Uterine Surgery should be published to the profession?*" If so, then the object was legitimate and praiseworthy. But if that was the object, why italicize as "*extraordinary*" this sentence: "*If convenient I would like to see her.*" In Prof. WHITE's first letter, to which mine was a reply, he says: "*should you wish an examination,* in case you advise excision, before undertaking it, *she can come down and see you,*" and it was only in response to this suggestion that I expressed a wish to see her. And in his second letter, in his reply to mine, he writes: "If my friend should be in Philadelphia during the coming year, *I will request her to visit you, &c.*" Now, let me ask again, why distinguish this sentence? Besides, it is brought up again in your criticism in such an offensive way, and the general tone of your criticism is so significant, that the object of publication would appear to be, not so much a matter of science, as an attempt at personal obloquy. I am perfectly willing, however, in this instance, to let that fall on whom it may.

I will enclose to you copies of both letters of Prof. WHITE to me, and, as they are gentleman-like in tone, I hope he will consent to their publication, allowing me the same liberty of italicizing sentences, that he assumed in the publication of my letter:

"Buffalo, Aug't 16. 1869.

"Dr. W. L. ATLEE:

Dear Sir,—I have a friend 45 years old, widow, who has never been pregnant, in pretty good health, who has a fibrous tumor, *probably intra-mural, of ten or twelve years* standing, and now extending above the umbilicus. It is irregular in form, but I find *no evidence in manipulating that there are adhesions, although she has suffered a good deal with superficial abdominal cramps and*

*pains*—no hemorrhages or leucorrhea. *The uterus is considerably enlarged, as shown by the sound, and crowded anteriorly.*

My object in writing is, in her behalf, to ask if you would advise extirpation ? If you would like any further or more minute description, I shall be happy to furnish it. *Should you wish an examination*, in case you advise excision, before undertaking it, *she can come down and see you.* Mrs. F. is highly respectable and very well connected, and having a good home, would probably prefer the operation at home, in case you decide upon making it. In my opinion, *the uterus is so involved in the tumor* that it would require to be removed at the neck.

*Some friends of yours* have advised Mrs. F. that you are removing tumors of this character, and I write at her request *to ask if it is true ?*

Should you not remember me, I will take the liberty of referring you to my my friend, Dr. S. D. Gross,

Awaiting your response, and with great respect, I remain,

Your ob't serv't,

James P. White."

" P. S.—*Please enclose bill for fee for correspondence, and I will remit at once.*"

"Yours truly, J. P. White."

To this letter of Prof. White, mine, the "*remarkable letter*," published on page 66 of your Journal, was the answer. Now, before making a copy of his second letter, allow me to extract his statement at page 65 of the Journal, so that all may be compared :

" I should state that the letter is in reply to one addressed, a few weeks since, to Dr. Atlee, stating that I had a patient of respectability and *wealth*, who had been informed by some *lay* friends of his that he was in the habit of excising fibroid growths of the uterus. It was stated in my communication to him, that the patient was forty-four years old, had suffered with this growth for ten or twelve years, that *she was now as large as the woman at term*, (*weight of tumor estimated at twelve or fifteen pounds ;*) *that it was hard in character, and that I could not determine whether it was intra-mural or only involved the uterus externally, but that it was not within the cavity of the organ ;* that the patient suffered little in her general health, had no severe hemorrhages or *pains*, and was anxious to know if he would relieve her of her deformity, and that I wrote at her request."

The second letter, in reply to mine, is in order :

"Buffalo, Aug. 25th, 1869.

" Dear Sir,—Your letter of the 17th inst. is received.

*I entirely concur with the opinion that an operation is not advisable.* Indeed I

have always insisted with my patient that it was not a suitable tumor for removal, *involving, as it doubtless does, the uterus and its appendages.* Is it possible, my dear doctor, that you can expect muriate of ammonia to "disperse" a fibroid of *ten or fifteen years* standing, and which has attained a size of *twelve or fifteen pounds ?*

My own experience would scarcely induce me to hope, by *medication to promote the absorption of a much smaller tumor*, especially if, as in this case, *all the most reliable remedies had been already faithfully used.*

Indeed my own experience and observation, both at home and abroad, had not induced me to suppose that the tumor which I described was removable. *It certainly could only be extirpated with the uterus.* My patient had, however, been much annoyed by being assured by some excellent friends (lay friends) of yours, *that you had frequently removed tumors of that character.* At her request, therefore, I wrote to ask the question, *well knowing the impracticability of the operation.* I am greatly obliged for your prompt reply and very moderate fee, which please find enclosed. If my friend should be in Philadelphia during the coming year. I will request her to visit you, and shall feel greatly obliged for any further suggestions in the case. In much haste. I am,

Truly yours,

JAMES P. WHITE."

WASHINGTON L. ATLEE, M. D.,
Philadelphia.

In my letter, to which this is a reply, *I gave no opinion upon the propriety of an operation in this case. I could not do so without a personal examination. I also stated that I do remove certain Uterine Fibroids by the knife.* So that the word "*concur*" in his letter does not quite apply.

In both the letters of Prof. WHITE the italics are my own.

Now, Sir, read the whole correspondence between Prof. WHITE and myself, and I am sure that no *gentleman* can object to the tenor of it, or its perfectly respectful character. Prof. WHITE and I have a right to differ in our opinions, but that difference does not allow to you or to him the privilege of attempting to degrade a professional brother. If he has had as large an experience in the treatment of Uterine Fibroids as I have had with the muriate of ammonia, then I consider it very "*extraordinary*" that it does not tally with my own, and if he has had no experience with it, then I consider it still more "*extraordinary*" that he should question the *fact* as stated by me. I have also "*faithfully used all the most reliable remedies,*" and have long since settled down on *muriate of ammonia* as the only one deserving of any confidence. After a practice of forty years, with an experience in pelvic and abdominal surgery, perhaps, not inferior to any member of the profession in the United States, I am ready to reiterate every word in that "*remarkable*" letter to Prof. WHITE; and let me further say, that as Mrs. F. has paid my fee for consultation, she is entitled to the benefit of such treatment until the propriety of an operation is decided upon.

With regard to the criticism, which follows the publication of my letter to Prof. WHITE, it is unworthy of notice. Before making another attempt at inditing so much nonsense, let me advise the critic to read up on the literature, and study the ethics, of his profession. For this advice, as it is to a member of our fraternity, *I charge no fee.*

WASHINGTON L. ATLEE,
1408 Arch Street.

Philadelphia, October 5th, 1869.

